# Comparative characterization of microRNA-71 of *Echinococcus granulosus* exosomes[Fn FN1]

**DOI:** 10.1051/parasite/2023060

**Published:** 2023-12-11

**Authors:** Lujun Yan, Yating Li, Rui Li, Mengqi Liu, Xuedong He, Xing Yang, William C. Cho, Mazhar Ayaz, Omnia M. Kandil, Yongchun Yang, Houhui Song, Yadong Zheng

**Affiliations:** 1 Key Laboratory of Applied Technology on Green-Eco-Healthy Animal Husbandry of Zhejiang Province, Zhejiang Provincial Engineering Laboratory for Animal Health Inspection & Internet Technology, Zhejiang International Science and Technology Cooperation Base for Veterinary Medicine and Health Management, China-Australia Joint Laboratory for Animal Health Big Data Analytics, College of Animal Science and Technology & College of Veterinary Medicine of Zhejiang A&F University Hangzhou 311300 China; 2 College of Animal sciences (College of Bee Science), Fujian Agriculture and Forestry University, Fujian-Taiwan Key Laboratory of Animal Pathogen Biology Fuzhou 350002 China; 3 Department of Medical Microbiology and Immunology, School of Basic Medicine, Dali University Dali 671000 Yunnan China; 4 Department of Clinical Oncology, Queen Elizabeth Hospital Hong Kong SAR China; 5 Cholistan University of Veterinary and Animal Sciences Bahawalpur 73000 Pakistan; 6 Depterment of Parasitology and Animal Disease, Veterinary Research Institute, National Research Centre Giza 12622 Egypt

**Keywords:** *Echinococcus granulosus*, Protoscolex, egr-miR-71, Exosome

## Abstract

Cystic echinococcosis (CE) is a global zoonotic disease caused by *Echinococcus granulosus*, posing a great threat to human and animal health. MiRNAs are small regulatory noncoding RNA involved in the pathogenesis of parasitic diseases, possibly *via* exosomes. Egr-miR-71 has been identified as one of the miRNAs in the blood of CE patients, but its secretory characteristics and functions remains unclear. Herein, we studied the secretory and biological activity of exosomal egr-miR-71 and its immunoregulatory functions in sheep peripheral blood mononuclear cells (PBMCs). Our results showed that egr-miR-71 was enriched in the exosome secreted by protoscoleces with biological activity. These egr-miR-71-containing exosomes were easily internalized and then induced the dysregulation of cytokines (IL-10 and TNF-α), nitric oxide (NO) and key components (CD14 and IRF5) in the LPS/TLR4 pathway in the coincubated sheep PBMCs. Similarly, egr-miR-71 overexpression also altered the immune functions but exhibited obvious differences in regulation of the cytokines and key components, preferably inhibiting proinflammatory cytokines (IL-1α, IL-1β and TNF-α). These results demonstrate that exosomal egr-miR-71 is bioactive and capacity of immunomodulation of PBMCs, potentially being involved in immune responses during *E. granulosus* infection.

## Abstract (fr)

L’échinococcose kystique (EK) est une maladie zoonotique mondiale causée par *Echinococcus granulosus*, représentant une grande menace pour la santé humaine et animale. Les miARN sont des petits ARN régulateurs non codants impliqués dans la pathogenèse des maladies parasitaires, éventuellement *via* les exosomes. Egr-miR-71 a été identifié comme l’un des miARN présents dans le sang des patients atteints d’EK, mais ses caractéristiques et fonctions sécrétoires restent floues. Ici, nous avons étudié l’activité sécrétoire et biologique du egr-miR-71 exosomal et ses fonctions immunorégulatrices dans les cellules mononucléées du sang périphérique (CMSP) de mouton. Nos résultats ont montré qu’egr-miR-71 était enrichi dans l’exosome sécrété par les protoscolex ayant une activité biologique. Ces exosomes contenant egr-miR-71 ont été facilement internalisés et ont ensuite induit la dérégulation des cytokines (IL-10 et TNF-α), de l’oxyde nitrique (NO) et des composants clés (CD14 et IRF5) de la voie LPS/TLR4 dans les CMSP de mouton co-incubées. De même, la surexpression d’egr-miR-71 a également modifié les fonctions immunitaires mais a montré des différences évidentes dans la régulation des cytokines et des composants clés, inhibant de préférence les cytokines pro-inflammatoires (IL-1α, IL-1β et TNF-α). Ces résultats démontrent que l’egr-miR-71 exosomal est bioactif et possède une capacité d’immunomodulation des CMSP, potentiellement impliquée dans les réponses immunitaires lors d’une infection à *E. granulosus*.

## Introduction

Cystic echinococcosis (CE) is one of the neglected tropical diseases by WHO classification and is caused by *Echinococcus granulosus* [1]. CE is also a serious zoonotic disease posing a great threat to human and animal health across the world. In the life cycle of the parasite, sheep act as a main intermediate host, while humans are occasionally infected due to consumption of egg-contaminated food or water. CE has a long latent period of 5–10 years, and patients with CE are currently treated only with surgical resection or/and with chemical drugs [5]. The lack of effective treatment approaches is partially attributed to poor understanding of the pathogenesis.

Exosomes are one class of extracellular vesicles with a diameter of 50 nm–150 nm and are derived from multivesicular bodies (MVBs) with a phospholipid bilayer membrane [40]. Exosomes are carriers to transport a great number of cargos including lipids, proteins, metabolites, DNA, and RNA (mRNA, microRNAs (miRNAs), and lncRNAs), which are actively involved in intercellular communication [34]. Recently, an increasing number of studies have demonstrated that parasite-derived exosomes are regulators of type II immune responses or other bioprocesses beneficial for survival [4, 22, 37]. In addition, it has been reported that miRNAs, as an important component in parasite-derived exosomes, regulate the production of immune response-related factors in the host to influence parasitism. However, the immune regulatory functions of miRNAs in the exosomes derived from *E. granulosus* remain largely unexplored.

MiRNAs, a type of non-coding small RNA of 18–22 nt in length, mainly engage in post-transcriptional regulation of gene expression by completely or partially complementing with the 3′ untranslated region (UTR) of target genes [11, 42]. An increasing number of miRNAs have been reported to play an important role in various biological and pathological processes, including development, immune response, and drug resistance [16, 23, 24]. miR-71 is one of the highly conserved miRNAs in helminths, including cestodes, trematodes, nematodes, and planarians [14]. Currently, miR-71 was found to be associated with development, neural synaptic activity, and longevity of helminths [12, 28, 30]. Most recently, nematode miR-71 was shown to regulate interferon regulatory factor 4 to modulate cytokine production, suggesting a potential ability of immunomodulation in host-parasite interactions [35]. In a previous study, we also observed that *E. multilocularis* miR-71 influenced the immune responses possibly *via* inhibiting the NO production in macrophages and *E. granulosus* miR-71 (egr-miR-71), one of the top five miRNAs expressed in the hydatid fluid-derived exosomes [43], and was shown to promote the migration and invasion of sheep peripheral blood mononuclear cells possibly *via* macrophage migration inhibitory factor [18], suggesting a potential role in immune responses. Most recently, egr-miR-71 was shown to be present in the blood of CE patients [2]. However, the role of circulating egr-miR-71 in exosomes remains unknown.

Toll-like receptor 4 (TLR4) is primarily expressed in defensive cells, such as mononuclear macrophages and endothelial cells, and recognizes conserved molecules in the evolution of pathogenic microorganisms, such as lipopolysaccharides (LPS), peptidoglycan, yeast polysaccharides, and nucleic acids of microorganisms. TLR4 mediates two signaling pathways: the MyD88-dependent and -independent pathways. Activation of both pathways stimulates immune cells to produce cytokines such as IL-1β and TNF-α during innate immune response [8, 20]. Accumulating studies have shown that the LPS/TLR4 signaling pathway plays an important role in the pathogenesis of echinococcosis. Several miRNAs derived from *E. multilocularis* were demonstrated to induce the dysregulation of several key components in the LPS/TLR4 pathway [7, 13]. In addition, it was also shown that host miRNAs mediated immune responses by regulating the LPS/TLR4 pathway during *Echinococcus* infection [9, 41].

In this study, we revealed the secretory and immunomodulatory characteristics of egr-miR-71 in *E. granulosus*-derived exosomes. We found that egr-miR-71 was indeed loaded into the exosomes with biological activity, which were easily internalized by sheep PBMCs. Both egr-miR-71 and egr-miR-71-containing exosomes induced the dysregulation of NO, cytokines and the LPS/TLR4 signaling pathway in sheep PBMCs, but seemed to exhibit different immune regulatory functions. These results provide a clue to further investigate the role of egr-miR-71 in the interplay between sheep and *E. granulosus*.

## Materials and methods

### Ethics statement

Animal experiments were approved by the Ethics Committee of Zhejiang Agriculture and Forestry University. Animals were treated strictly according to the Good Animal Practice Requirements of the Animal Ethics Procedures and Guidelines of the People’s Republic of China.

### Parasite

Cyst masses of G1 genotype *E. granulosus* were dissected from the liver of naturally infected sheep slaughtered in an abattoir in the Xinjiang Autonomous Region, China. The hydatid fluid was carefully collected with a sterile syringe and then protoscoleces in the hydatid fluid were obtained by three rounds of gravity sedimentation, as previously reported [39]. Subsequently, the protoscoleces were checked for viability using Trypan blue and 50,000 protoscoleces were incubated in T75 cell culture flasks (Corning Inc., Corning, NY, USA) with 15 mL of FBS-free RPMI 1640 (Gibco, Waltham, MA, USA).

### Cell culture and transfection

Peripheral blood mononuclear cells (PBMCs) were isolated from three healthy sheep by polysucrose-diammonia density gradient centrifugation, according to the instructions of the manufacturer (TMD Science) [25]. The three PBMC samples were washed in 3× volumes of pre-chilled phosphate-buffered saline (PBS) before inoculation into cell culture flasks, followed by checking their viability by Trypan blue exclusion (Sigma-Aldrich, St. Louis, MO, USA). Each sample was set in triplicate and the experiments were independently performed three times.

For electric transfection, approximately 1 × 10^6^ cells were resuspended in Entranster TME Electroporation Solution (Engreen). 1.25 μL of egr-miR-71 mimics (100 nM; Invitrogen Waltham, MA, USA), an artificially-synthesized analog of egr-miR-71, or negative control construct (100 nM, Invitrogen) were added into each 100 μL of the electroporation buffer, mixed and added into an electroporation cuvette (4 mm gap), followed by electroporation at 150 V, 25 μF, 50 Ω for 6 ms. Samples after electroporation were immediately incubated in RPMI-1640 with 100 ng/mL LPS (Sigma) and 10 ng/mL IFN-γ (R&D). Cell supernatant was collected at 12 h and 24 h after transfection for later use.

### Separation of exosomes derived from *E. granulosus* protoscoleces

After incubating protoscoleces in FBS-free RPMI 1640 for 24 h, the medium was collected and centrifuged at 200× *g* for 10 min at 4 °C, then the supernatant was centrifuged at 2,000× *g* for 20 min and finally centrifuged at 10,000× *g* for 40 min at 4 °C. The supernatant was filtered using a 0.22 μm filter and the filtrate was centrifuged at 110,000× *g* for 2 h at 4 °C. The precipitate was washed in filtered PBS and re-centrifuged as above. Then, the exosome-containing pellets were stored at −80 °C for later use.

### Transmission electron microscopy and nanoparticle tracking analysis

The enriched exosomes were diluted with filtered PBS, employed onto a 2 nm copper grid and stained using phosphotungstic acid, as previously described. Transmission electron microscopy (TEM, JEOL, Japan) was used to observe the morphology of the exosomes. The size distribution of the exosomes was detected using Particle Metrix ZetaView^®^ nanoparticle tracking analysis (NTA) technology (Malvern Panalytical, Malvern, England, UK).

### Western blotting

Exosomes (20 mg), protoscoleces (20 mg), and PBMCs were treated by RIPA lysis buffer to extract proteins, and then 10% SDS-PAGE gel electrophoresis was used to separate the proteins, followed by Western blotting. Briefly, polyvinylidene fluoride membranes were used for protein transfer. After incubation with 5% bovine serum albumin (Sigma), the membranes were treated overnight at 4 °C with mouse anti-enolase (1:1000), rabbit anti-14-3-3 (1:1000, previously prepared in our lab), rabbit anti-TIRAP (1:500; ABclonal, Woburn, MA, USA) and rabbit anti-tubulin antibodies (1:2,000; Abmart, Berkeley Heights, NJ, USA), respectively. And then, the membranes were incubated for 1 h at room temperature with suitable HRP-conjugated antibodies: goat anti-mouse IgG antibodies (1:10,000; SeraCare Life Sciences, Milford, MA, USA) or goat anti-rabbit IgG antibodies (1:10,000, SeraCare Life Sciences). Finally, signals were visualized on X-ray films or Image Lab software (Bio-Rad, Hercules, CA, USA) using enhanced chemiluminescence (ThermoFisher Scientific, Waltham, MA, USA).

### Extraction of total RNA and Real-time RT-PCR

Total RNA were extracted from *E. granulosus*, exosomes and PBMCs by Trizol reagent according to the following method. The samples were treated by 1 mL of Trizol reagent and gently mixed by pipette. Then 3.5 μL of cel-39-5p spike-in control with a concentration of 1.6 × 10^8^ copies/μL were added as an external reference, followed by chloroform extraction. After standing 10 min at RT, samples were centrifuged at 12,000× *g* for 10 min, and the upper aqueous phase was transferred into a new tube. A total of 10 μg of glycogen were added and mixed well, followed by addition of 500 μL of isopropanol and incubation at −20 °C for 10 min. After centrifugation at 12,000× *g* for 10 min, the pellets were washed using 75% ethanol and centrifuged at 7,500× *g* for 10 min at 4 °C. The total RNA samples were resolved in nuclease-free water and their integrity and concentration were analyzed by formaldehyde-denaturing electrophoresis and Nanodrop 2000 (ThermoFisher Scientific), respectively.

For miRNA and mRNA reverse transcription, 500 ng of total RNA were used to synthesize first-strand cDNA by miRNA First-strand cDNA Synthesis Kit (GeneCopoeia, Rockville, MD, USA) and RevertAid First Strand cDNA Synthesis Kit (ThermoFisher) according to the manufacturer’s method, respectively. Then, the real-time qPCR was performed using All-in-one qPCR Kit (GeneCopoeia) by ABI 7500 system, according to the manufacturer’s instructions with the following steps: 95 °C for 10 min, followed by 40 cycles of 95 °C for 10 s, and 60 °C for 1 min. To calculate the relative expression levels of miRNA and mRNA, cel-39-5p and β-actin were used as a reference in these experiments, respectively. Each sample was tested in triplicate and the relative expression level of target genes were calculated by 2^−ΔΔCt^ method. All specific primers used are listed in Table 1.

**Table 1:** qPCR primers for key components in the TLR4 signaling pathway, cytokines and egr-miR-71.

Gene symbol	Accession number^1^	Gene name	Sequence (5′–3′)	Size (bp)
*GAPDH*	NM_001190390.1	Glyceraldehyde-3-phosphate dehydrogenase	F: CTAGGCTACACTGAGGAC	74
			R: CAGCATCGAAGGTAGAAG	
*TLR4*	NM_001135930.1	Toll like receptor 4	F: GTATCTCTAGATGACTTCCC	11
			R: CAGGTTGGGAAGGTCAGAAA	7
*CD14*	NM_001077209.2	CD14 molecule	F: CACCAAGACGACCCGATGAT	16
			R: AGGGCGATCTGAGCCAATTC	1
*MYD88*	XM_042235740.1	Myeloid differentiation factor 88	F: TAGGGCAAAAGCCTGAGTATT	11
			R: ACAACTTCAGCCGATAGTTTG	4
*TIRAP*	XM_042238388.1	TIR domain containing adaptor protein	F: GCTCAGAGGTGTCTCCCATCCC	10
			R: TGGCACACGCACACATCATAGG	9
*TICAM2*	XM_004010183.5	TIR domain-containing adapter molecule 2	F: GACGTCAGATTCCAAGCAAT	12
			R: CTTCTTCTGTGTCCTCTTCA	8
*IRF3*	XM_004015378.5	Interferon regulatory factor 3	F: GAAGGAAGTGTTGCGTTTAGC	12
			R: TGTCTGCCATTGTCTTGAGC	9
*IRF5*	XM_042248802.1	Interferon regulatory factor 5	F: AATCCTAGTTCTAGTCTCCC	13
			R: TTCATTGGAGGTGAGTCGTG	1
*AP-1*	XM_034503172.1	Transcription factor AP-1	F: TGAAGGAAGAGCCGCAGAC	24
			R: CCACCTGTTCCCTGAGCATA	3
*RIPK1*	XM_027958685.2	Receptor interacting serine/threonine kinase 1	F: AACAGAAGGTGCAGTACCAT	15
			R: AGGTCAGCTATCTCTGAACA	3
*RELA*	XM_027959295.2	RELA proto-oncogene	F: TCTCATCCCATCTTTGACAACC	13
			R: TGTCCTCTTTCTGCACCTTGT	3
*RELB*	XM_015100238.3	RELB proto-oncogene	F: TGGCCTTCCAATCAGGATA	14
			R:AAATGAGCTCAGGAGAAACC	1
*IL-1α*	NM_001009808.1	Interleukin 1 alpha	F: GCCAATGATACCGAAGAAGA	13
			R: ATACTTTGATTGAGGGCGTC	1
*IL-1β*	NM_001009465.2	Interleukin 1 beta	F: CATCCTTTCATTCATCTTCG	12
			R: GATTTTTGCTCTCTGTCCTG	0
*IL-6*	NM_001009392.1	Interleukin 6	F: TCAGTCCACTCGCTGTCTCC	10
			R: TCTGCTTGGGGTGGTGTCAT	7
*IL-10*	NM_001009327.1	Interleukin 10	F: GGTGATGCCACAGGCTGAGAAC	14
			R: GCTCCACCGCCTTGCTCTTG	3
*IL-11*	XM_027978781.2	Interleukin 11	F: ACGGAGACCACAGCCTGGATTC	13
			R: CAGCCACTGCACATGCCTCAG	4
*IL-13*	NM_001082594.1	Interleukin 13	F: CCTGATCAGCATCTCCAACTG	11
			R: TTGGTGTCTCGGACGTACT	7
*IL-17*	XM_004018887.5	Interleukin 17	F: GCGTCTATGAGAACTGCCTCTATGT	11
			R: AGTTCTTGTCCTCAGTAGGTGGGCA	8
*TNF-α*	NM_001024860.1	Tumor necrosis factor alpha	F: ATGTGGAGCTGGCGGAGGAG	12
			R: GCAGGCAGAAGAGCGTGGTG	6
*IFN-β*	XM_027963972.2	Interferon beta	F: GGCAGTTACCTTCAACT	12
			R: CTGGAGCATCTCATACATG	8
*iNOS*	XM_042255454.1	Inducible nitric oxide synthase	F: ATCAAATCCCAAAAGGTGGAC	17
			R: TCTGGAGATGTCTTTACCGT	6
egr-miR-71	/		GCGAGCACAGAATTAATACGACTCACTATAGG(T)_12_VN^2^	
			F: TGAAAGACGATGGTAGTGAGA	
			R: GCGAGCACAGAATTAATACGAC	

1 The accession numbers are for the genes deposited in NCBI.

2 The primer used for reverse transcription. “V” stands for A, G or C, and “N” for A, T, G or C.

“/”: not applicable.

### Exosome internalization

Exosomes were labeled with PKH32 Red Fluorescent Cell Linker Kit (Sigma Aldrich), according to the manufacturer’s instructions. For exosome internalization, 20 μg of PKH26-labeled exosomes were added into sheep PBMC cells. After incubation for 24 h, flow cytometry (Merck, Rahway, NJ, USA) and laser scanning confocal microscopy (Leica, Germany) were used to evaluate internalization efficiency of the exosomes.

### Construction of recombinant plasmid and egr-miR-71 functional activity detection

The 3′ UTR fragments of the wild type (Wt) and mutant type (Mut, containing point mutations in the egr-miR-71 binding site) of Nemo-like kinase (NLK), a target of egr-miR-71 validated in a previous study, were amplified by PCR (F: 5′–CGGAATTCTTCGACAAGCTGCTTCCGTGTACGT–3′ and R: 5′–CC*CTCGAG*TAGAAATGAAATGAGTCACGGCGTG–3′ for Wt, and F: 5′–CGGAATTCTTCGACAAGCTGCTTCCGTGTACGT–3′ and R: 5′–CC*CTCGAG*TAGAAATGAAATGAGTCACGGCGTG–3′ for Mut). Then amplicons were subcloned into the pCMV-eGFP vector to generate bioactive-reporter constructs. All the constructs were verified by sequencing and PCR. Then 100 ng of the bioactive-reporter plasmid was transiently transfected into HEK-293T cells cultured in 24-well plates using Lipofectamine 2000 (Invitrogen), according the manufacturer’s instructions, and 1.25 μL of egr-miR-71 mimics and negative control (100 nM, ThermoFisher Scientific) were later transfected by RNAimax (ThermoFisher Scientific), according to the manufacturer’s instructions, respectively, followed by incubation with the exosomes. After 36 h, the mean fluorescence intensity (MFI) of cells was detected by flow cytometry (Guava^®^ EasyCyte, Merck, Darmstadt, Germany).

### Determination of NO and enzyme-linked immunosorbent assay (ELISA)

Following sheep PBMCs treated with egr-miR-71 and exosomes, the culture supernatant was collected to detected the levels of nitric oxide (NO) using Griess reagent (Invitrogen), as previously described [43] and tumor necrosis factor-α (TNF-α) by Double Antibody Sandwich ELISA Kit (JL, China) according to the instructions of the manufacturer. For ELISA, 100 μL of supernatant and standards were added into each well and incubated at 37 °C for 2 h. After multiple washes, 100 μL of biotin (1×) were added into each well and incubated at 37 °C for 1 h. After washing, HRP-avidin, TMB substrate and stop solution were sequentially added. Finally, OD values at 570 nm and 450 nm were recorded using a microplate reader (Bio-Rad). Each sample and standard were set in triplicate.

### Statistical analysis

GraphPad Prism 5 software was used for data analysis. Statistical significance was analyzed using an unpaired Student’s *t*-test for comparison of two groups and ANOVA for comparison of three groups or more. *p*-values not more than 0.05 were considered to be statistically significant.

## Results

### Identification of *E. granulosus*-derived exosomes

**Figure 1:**
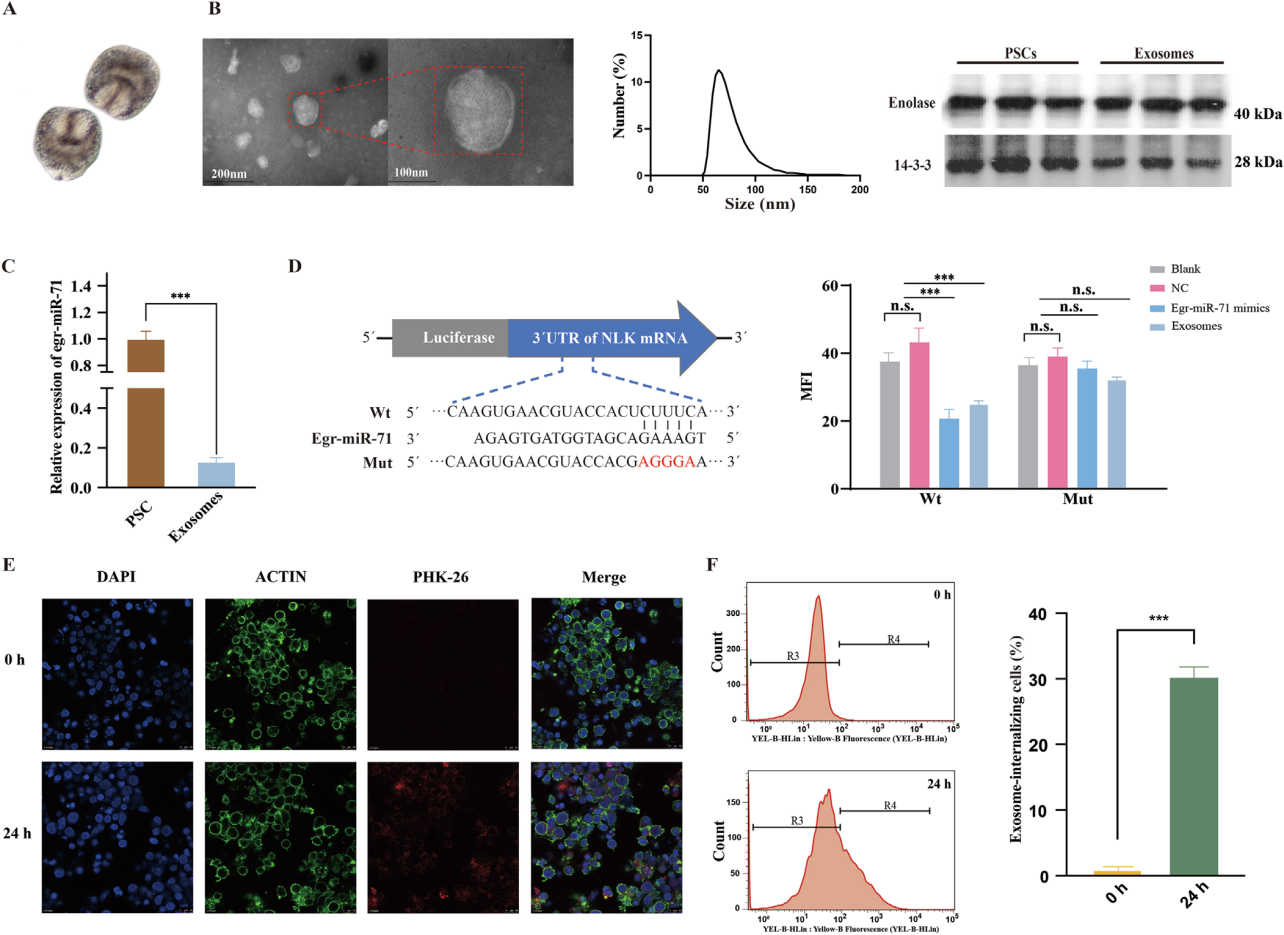
Identification of egr-miR-71 in the exosomes secreted by *E. granulosus* protoscoleces. (A) Purified protoscoleces. (B) TEM observation of the enriched exosomes (left), NTA analysis of the size of the exosomes (middle) and Western blotting analysis of the exosomes (right). (C) Detection of the abundance of egr-miR-71 in exosomes by reverse transcription-PCR. (D) Detection of biological activity of egr-miR-71 in exosomes. (E) Internalization of the exosomes by sheep PBMCs observed by confocal microscopy. (F) Flow cytometry analysis of the exosome-internalizing sheep PBMCs. Data are expressed as mean ± SD; ***p < 0.001. Data for the final analysis are from three independent experiments. PSC, protoscoleces; NLK, Nemo-like kinase; Wt, a wild type of the NLK 3’UTR; Mut, a mutant type of the NLK 3’UTR; MFI, mean fluorescence intensity; n.s., not significant; NC, negative control.

To investigate the potential functions of egr-miR-71 in host-parasite interactions, we collected the protoscoleces and cultured them in serum-free medium (Fig. 1A). Using differential centrifugation, the exosomes were isolated and their morphology was observed using transmission electron microscopy. The exosomes were shown to be vesicles enveloped by membranes and the size was between 50 nm and 120 nm (Fig. 1B). In addition, both the exosomal biomarkers (Enolase and 14-3-3) were also presented in the exosomes (Fig. 1B).

### Enrichment and biological activity of egr-miR-71 in *E. granulosus*-derived exosomes

To explore a role of egr-miR-71 in the exosomes, we assessed its abundance and functional activity by qPCR and luciferase reporting system, respectively. It was found that egr-miR-71 was loaded into the exosomes, but the abundance was significantly lower than that in protoscoleces (*p* < 0.01, Fig. 1C).

Then the 3′ UTR of NLK was used to construct a luciferase reporter system. Using flow cytometry, we detected the MFI in the HEK-293T cells. The MFI in the egr-miR-71 mimics group and exosome group was significantly decreased compared with that in the NC group in the cells transfected with the Wt constructs (*p* < 0.01, Fig. 1D). However, this decrease was not significant in the cells transfected with the Mut constructs (*p* < 0.01, Fig. 1D). The data demonstrate that both egr-miR-71 mimics and egr-miR-71 in the exosomes effectively bind to the 3’UTR of NLK, suggesting that egr-miR-71 in the exosomes is biologically active.

### Internalization of *E. granulosus*-derived exosomes by PBMCs

To confirm whether the *E. granulosus*-derived exosomes are internalized by the host cells, we pre-labeled the exosomes with PKH-26, co-incubated them with sheep PBMCs and then analyzed the internalization efficiency. Using laser confocal microscopy, we found that fluorescence signal was significantly increased in the PBMCs incubated with exosomes after 24 h (Fig. 1E). Furthermore, we also assessed the internalization efficiency of *E. granulosus*-derived exosomes by flow cytometry. The results showed that the fluorescence signal existed in approximately 30% of total PBMCs after being treated with exosomes (Fig. 1F). This result suggests that the *E. granulosus*-derived exosomes are easily internalized by sheep PBMCs.

### Effects of *E. granulosus*-derived exosomes on immune functions of sheep PBMCs

**Figure 2:**
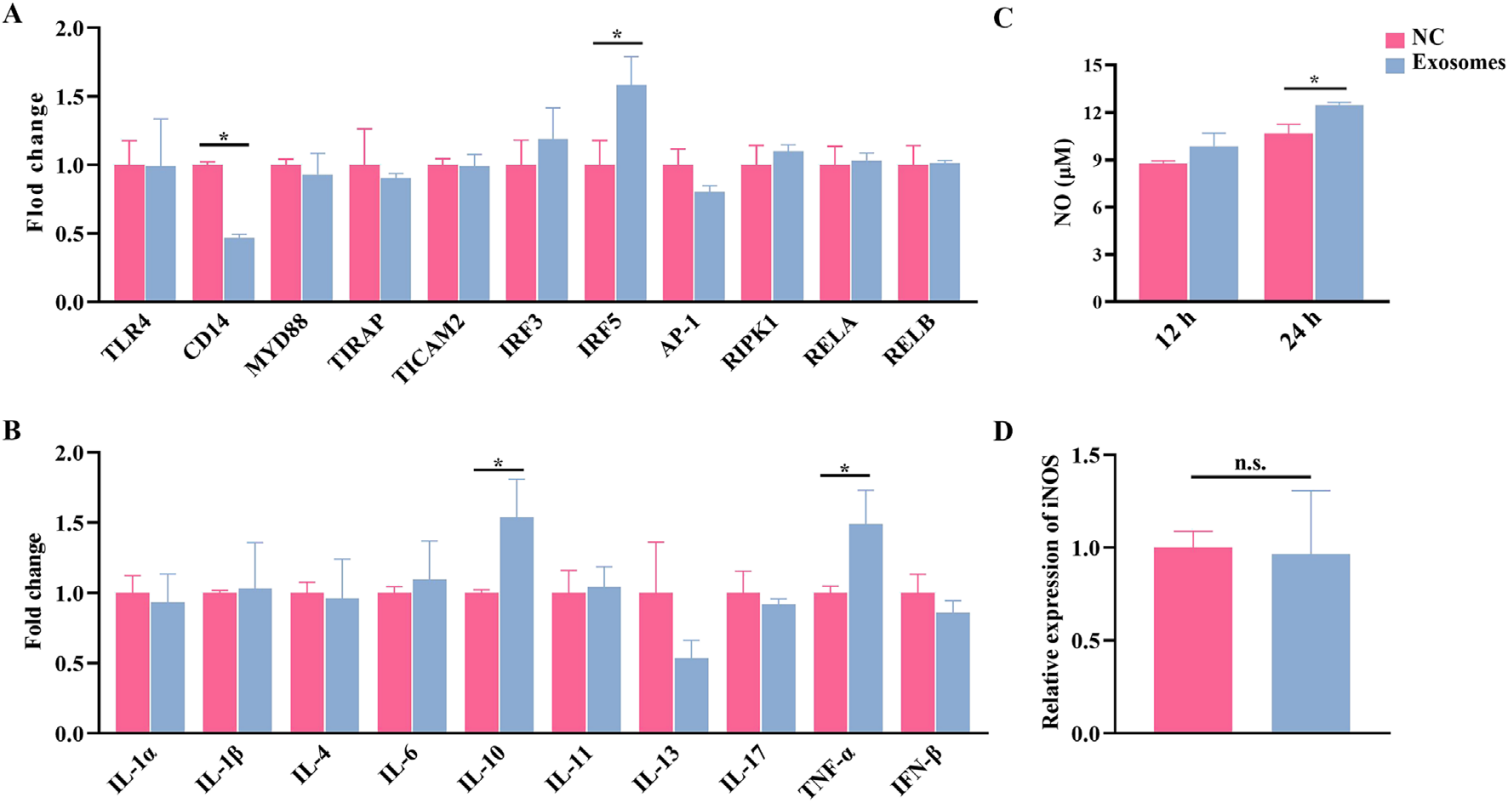
Regulatory effects of exosomes on immune functions of sheep PBMCs. (A) qPCR analysis of the expression of the core components in the LPS/TLR4 pathway after treatment with exosomes. (B) qPCR analysis of the expression of cytokines after treatment with exosomes. (C) The level of NO after treatment with exosomes determined by Griess reagent. (D) qPCR analysis of the expression of the iNOS after treatment with exosomes. Data are expressed as mean ± SD; *p < 0.05. Data for the final analysis are from three independent experiments. NC, negative control.

To determine whether egr-miR-71 is a main ‘virulent factor’ of the *E. granulosus*-derived exosomes or not, we first evaluated their potential effects on immune functions of PBMCs. The results showed that exosomes significantly disturbed the expression levels of CD14 and IRF5 (*p* < 0.01, Fig. 2A). In addition, the levels of IL-10 and TNF-α were significantly increased after treatment (*p* < 0.05), whereas the level of IL-13 was slightly reduced (Fig. 2B). Furthermore, the results showed that the production of NO was significantly increased in exosome-treated PBMCs at 48 h (*p* < 0.01, Fig. 2C). Inconsistently, the expression level of iNOS, a gene responsible for the synthesis of NO, remained constant in the exosome-treated PBMCs (Fig. 2D).

### Effects of egr-mir-71 on immune functions of sheep PBMCs

**Figure 3:**
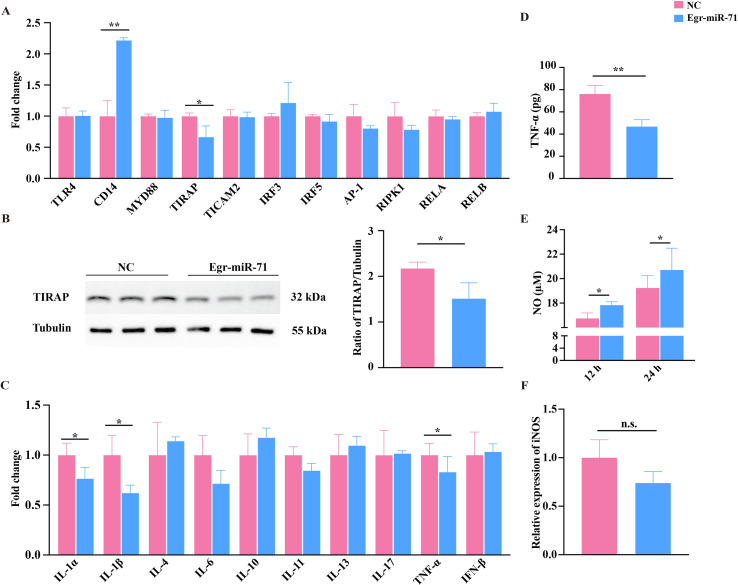
Regulatory effects of egr-miR-71 mimics on immune functions of sheep PBMCs. (A) qPCR analysis of the expression of the core components in the LPS/TLR4 pathway after transfected with egr-miR-71 mimics. (B) ELISA analysis of the expression of TIRAP after transfection with egr-miR-71 mimics. (C) qPCR analysis of the expression of cytokines after transfection with egr-miR-71 mimics. (D) Sandwich ELISA analysis of the level of TNF-α after transfection with egr-miR-71 mimics. (E) The level of NO after transfection with egr-miR-71 mimics determined by Griess reagent. (F) qPCR analysis of the expression of the iNOS after transfection with egr-miR-71 mimics. Data are expressed as mean ± sd; *p < 0.05, **p < 0.01. Data for the final analysis are from three independent experiments. NC, negative control; n.s., not significant.

To investigate the contributions of exosomal egr-miR-71 to exosome-induced immune modulation in sheep PBMCs, we then checked the expression of the above LPS/TLR4 pathway-related components and cytokines. The results showed that egr-miR-71 mimics significantly disturbed the expression levels of CD14 and TIRAP (*p* < 0.05, Fig 3A) and TIRAP was also verified to be decreased at the protein level in transfected PBMCs (*p* < 0.05, Fig 3B). Moreover, the levels of IL-1α, IL-1β and TNF-α markedly decreased in the PBMCs transfected with egr-miR-71 mimics compared with that in the NC group (*p* < 0.05, Fig. 3C). ELISA further confirmed the decreased TNF-α in response to egr-miR-71 overexpression (*p* < 0.01, Fig. 3D). In addition, the production of NO was remarkably increased in the PBMCs after being treated with egr-miR-71 mimics (*p* < 0.05, Fig. 3E), while the expression of iNOS was unchanged (Fig. 3F).

## Discussion

Egr-miR-71 is one of the highly expressed miRNAs in *Echinococcus* species and has been shown to be involved in protoscolex development [28]. In this study, egr-miR-71 was demonstrated to be loaded into the exosomes that were vesicles with a diameter of 50–150 nm and contained 14-3-3 and enolase, which is consistent with the results reported in other parasites [19].

Before exploring the role of egr-miR-71, we first checked whether exosomal egr-miR-71 had biological activity or not. The results demonstrated that both egr-miR-71 mimics and exosomes dramatically decreased the MFI in the 293T cells transfected with the Wt construct but not the Mut construct, suggesting that egr-miR-71 in the exosomes derived from *E. granulosus* is functionally active. Next, we proved that the exosomes were able to be easily internalized by sheep PBMCs. These results demonstrate that *E. granulosus* has a secretory pathway that releases large amounts of functionally active egr-miR-71 into host cells, such as PBMCs, *via* exosomes.

In treated sheep PBMCs, the exosomes were shown to affect the levels of two key components (IRF5 and CD14) in the LPS/TLR4 pathway. Furthermore, the exosomes significantly up-regulated a pro-inflammatory cytokine (TNF-α) and down-regulated an anti-inflammatory cytokine (IL-10). These similar dysregulations have been observed in sheep PBMCs incubated with the exosomes from *E. granulosus* hydatid fluid [39]. These results are also consistent with recent reports on host immune responses induced by exosomes secreted by other parasites. It has been shown that extracellular vesicles derived from *Toxoplasma gondii* significantly increase the levels of IL-10, TNF-α, and iNOS in murine macrophages [33]. Similarly, *Taenia pisiformis* exosomes participated in promoting macrophages to M2 polarization by affecting the expression of Arg-1, IL-4, IL-6, IL-10, IL-13, iNOS, IFN-γ, and IL-12 [37].

In contrast, egr-miR-71 seems to have a primary function to suppress immune response in sheep PBMCs. Our data showed that the levels of three pro-inflammatory cytokines (IL-1α, IL-1β, and TNF-α) were significantly down-regulated in PBMCs after egr-miR-71 treatment. Moreover, although CD14 was upregulated, TIRAP was downregulated in response to egr-miR-71 treatment. In the LPS/TLR4 pathway, as a putative LPS receptor, CD14 can recognize and combine with LPS to facilitate LPS binding with TLR4 [10, 29]. Subsequently, TIRAP acts as the first adaptor protein to bind with TLR4 at the plasma membrane and further recruits MyD88 to form a complex that promotes the production of inflammatory cytokines, such as IL-1, IL-6, TNF-α, and Type III interferons [17, 36]. These results indicate that egr-miR-71 inhibits the production of pro-inflammatory cytokines by down-regulating TIRAP.

It was also found that the expression of IRF3 and AP-1 was slightly up-regulated and down-regulated by both of exosomes and egr-miR-71, respectively, while the expression of IFN-β remains unchanged. As transcription factors, both IRF3 and AP-1 are activated by MyD88/TLR and translocated to the nucleus to bind to DNA, thus mediating the production of type I interferons and several inflammatory cytokines [15]. Of them, IRF3 is widely believed to be required by mammalian cells to mount an innate immune response against viruses. However, IRF3 seems to have an unexpected pro-parasitic role in supporting the replication of the parasite. A study reported that the replication of *T. gondii* was remarkably impaired in IRF3-deficient cells, and the interferon-stimulated genes induced by parasite-activated IRF3 was necessary, whereas type I interferons were not important [21]. Conversely, AP-1 has been found to be inactivated by *Leishmania* infection, which resulted in its reduced nuclear translocation in macrophages, suggesting that the inactivation of AP-1 is involved in survival in the host [6]. Therefore, it is worth pinpointing the roles of IRF3 and AP-1 in surviving harsh immune responses during infection.

In this study, it is clear that egr-miR-71 and egr-miR-71-loading exosomes exhibit an obvious difference in regulation of immune functions of sheep PBMCs. Exosomes contain a variety of bioactive molecules that play a crucial role in host-parasite interactions [31, 38]. It has been shown that the exosome derived from *E. granulosus* is particularly rich in heat shock proteins 70 and 14-3-3, which were reported to induce immune responses [3, 26, 27, 32]. The presence of a plethora of the bioactive molecules participating in immune responses may explain the incongruent immunoregulatory functions between exosomes and egr-miR-71 in sheep PBMCs.

In summary, we demonstrated a secretory pathway of bioactive egr-miR-71 that was transported by exosomes to host cells such as PBMCs, and that is involved in immunomodulation. This study provides a new direction to investigate the immunity functions of egr-miR-71 in parasite-host interactions.
